# Bond Performance of CFRP Strands to Grouting Admixture for Prestressed Structure and Development of Their Bond–Slip Constitutive Models

**DOI:** 10.3390/polym15132906

**Published:** 2023-06-30

**Authors:** Ce Wang, Shuai Guan, Md Sabbrojjaman, T. Tafsirojjaman

**Affiliations:** 1 CCCC Highway Consultants Co., Ltd., No. 33, Dongsiqiancaomian Alley 33, Dongcheng District, Beijing 100010, China; 2 The Key Laboratory of Urban Security and Disaster Engineering of Ministry of Education, Beijing University of Technology, Pingleyuan Road 100, Beijing 100124, China; 3 School of Architecture and Civil Engineering, The University of Adelaide, Adelaide 5005, Australia

**Keywords:** CFRP strand, grouting admixture, pull-out test, bond strength, constitutive model

## Abstract

Prestressed concrete structures have witnessed widespread use in building and infrastructure applications during the last two decades due to their high stiffness and strength indices. However, structural failures caused by the corrosion of steel reinforcing bars or strands have proliferated, opening the door for carbon fibre-reinforced polymer (CFRP) strands as an excellent alternative with high corrosion resistance. The bonding interaction between the CFRP strands and concrete is the fundamental parameter in shaping the structural behaviour of CFRP prestressed concrete structures. In this paper, the bonding behaviour between CFRP strands and concrete with grouting admixture is experimentally investigated based on three groups of standard pull-out tests. The bond strength of CFRP strands was systematically studied and compared against steel strands. The untreated CFRP strands exhibited an inefficient bonding strength with the grouting admixture, equivalent to only 5% compared to steel strands of the same diameter. Surface coating with epoxy quartz sand can significantly improve the anchoring efficiency of CFRP strands up to 14 times compared to the untreated strands, which is approximately as efficient as steel strands. Moreover, the bond–slip curves between CFRP strands and concrete were analysed and were found to be different compared to steel strands. Finally, this study proposed bond–slip constitutive models of CFRP strands with better applicability, using an exponentially damped sine function to fit the residual segment of the curve.

## 1. Introduction

The feasibility of applying fibre-reinforced polymer (FRP) bars and strands to prestressed concrete structures has received broad attention in the last few years due to the development of FRP materials, which are characterised by their excellent corrosion resistance. This attention was driven by the deterioration of structural serviceability and strength of prestressed concrete structures reinforced with steel strands, which are characterised by their serious corrosion problems. Moreover, these corrosion problems cause structural deficiency of the prestressed concrete structures in terms of service life and maintenance requirements [1]. Currently, the maintenance of steel corrosion includes repairing, strengthening, or replacing the damaged steel bars or wires in the structure, which adds huge economic costs and expenditures [2]. Therefore, researchers and engineers are investigating the use FRP bars and strands against steel to enhance the corrosion resistance of structural members in extreme environments [3,4,5,6,7]. This research studies the structural and bonding behaviour of carbon-fibre-reinforced polymer (CFRP) strands and their capability to replace steel strands in prestressed concrete structures with grouting admixture to improve the bonding performance.

The bonding interaction between the strands and concrete is the fundamental parameter in shaping the structural behaviour of prestressed concrete structures, as it is the key to the “cooperation” between the strand and concrete [8]. The anchoring performance and surface roughness are the two main factors affecting the interface performance between FRP bars and concrete. FRP bars are anisotropic materials and have different mechanical and physical characteristics compared to steel bars. The bond strength of FRP bars is characterised by several parameters, such as the bar diameter, bar shape, bar surface texture (smooth, sand-coated, ribbed, helically wrapped, or braided), embedment length, embedment length location, mechanical properties, and environmental conditions [9,10]. Park et al. [11] investigated the bond performance between FRP bars and concrete and studied the effect of fibre and surface types on the bond strength. FRP bars with sand coating exhibited superior bond strength compared to FRP spiral- or strand-type. According to Benmokrane [12] and Tighiouart [13], the bond performance decreases as the diameter of the rebar is increased.

The bond performance of FRP bars has been extensively investigated for both conventional and high-strength concrete [14,15,16,17]. Solyom et al. [18] proved experimentally that FRP bars can show a better bond strength, stiffness, and elastic modulus compared to steel bars. Moreover, the bond strength was found to increase when the concrete compressive strength is increased over 30 MPa. This finding on the increase in the FRP bar’s bond strength when increasing the concrete compressive strength was also documented and quantified by Achillides [19]. Olanitori et al. [20] concluded that the ultimate load and shear capacity of reinforced concrete beams decreases as the compressive strength of concrete decreases. The compressive strength of concrete significantly impacts the bar pull-out capacity near the end of the beam, as the bar pull-out capacity was found to decrease when the compressive strength is increased. According to Shima et al. [21], the bond strength of CFRP bars is proportional to 1/2–2/3 power of the concrete compressive strength in the zone of 38–84 MPa. On the other hand, Okelo et al. [22] proposed that the average bond strength of the FRP bars was proportional to 1/2 power of the concrete compressive strength in the zone of 29–60 MPa based on testing results. Understanding the bond performance and mechanism between FRP strands and concrete will facilitate design guidelines and recommendations to broaden the use of CFRP prestressed concrete structures.

Several comparative studies were conducted on the bonding properties of FRP bars and stranded wires and also compared the performance of FRP bars against traditional steel bars and strands in concrete [23,24,25]. The bonding behaviour of CFRP bars, GFRP bars, and steel strands was studied by Jun Zhao et al. [23] under different bonding lengths and different concrete strength conditions. The corresponding bond–slip curves were analysed, and the bonding strength of CFRP, GFRP, and steel strands was 55%, 78%, and 25%, respectively, compared to steel bars under the same conditions. Xue, Weichen et al. [24] compared the bonding properties of high-strength CFRP stranded wires in different binders such as grout, epoxy resin, high-performance concrete, and ordinary concrete through 48 pull-out tests. It was found that higher bonding strength can be developed through higher compressive strength of concrete, while the bonding strength of CFRP stranded wire is 1.3 to 1.4 times higher than that of steel strands under the same circumstances. It is a common practice to wrap the surface of FRP bars with ribbed or epoxy quartz sand to increase the friction between the reinforcement and concrete. Thus, it is necessary to analyse the bonding performance between the sticky sand-stranded CFRP wire and concrete to quantify the effect of surface-coating type on the bond strength.

The Bertero–Popov–Eligehanusen (BPE) model proposed by Eligheuasen et al. [26] is one of the well-known analysis models of bond–slip behaviour of reinforcement to concrete. This model is divided into an ascending segment, platform segment, and descending segment and is obtained from the bond strength–slip (τ–*s*) curve of steel reinforcement and concrete, which is different from the behaviour of FRP reinforcement and concrete. Therefore, several models were established and adjusted to be applicable to FRP bars through experimental and theoretical studies; these include the improved BPE model, Cosenza–Manfredi–Realfonzo (CMR) model [27], continuous curve model, etc.

**Figure 1:**
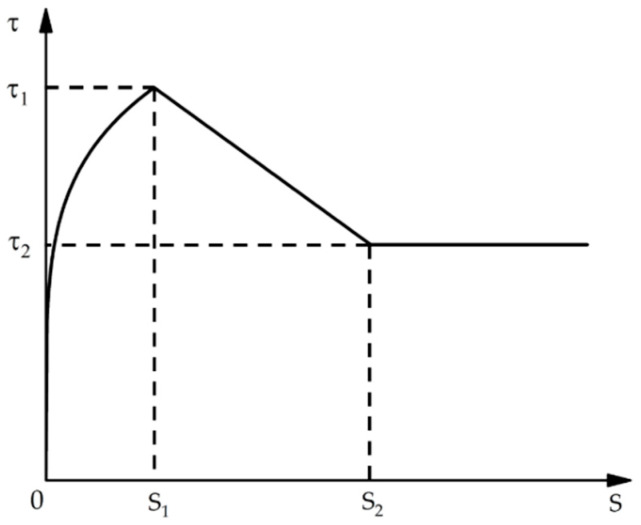
Typical bond–slip curves of the modified BPE model with different strength and slip values.

In 1996, Cosenza et al. [27] found that the bond–slip curve of FRP bars is not characterised by a platform segment. Thus, the original BPE model was adjusted to propose the modified BPE model for the bond–slip analysis of FRP bars, as shown in Figure 1. The model has the following shortcomings: (1) The model curve is not smooth and continuous at the peak bond strength point. (2) The descending section is a straight line, which is not consistent with the experimental phenomenon. (3) The residual bond stress is a constant, which cannot reflect the experimental fluctuation of the residual bond strength of FRP bars.

The modified BPE model is expressed as follows:

(1-1) $$\frac{\tau }{\tau_{1}}={\left(\frac{s}{s_{1}}\right)}^{\alpha },s<s_{1}$$

(1-2) $$\tau =\tau_{1}(1+p-\frac{ps}{s_{1}}),s_{1}<s\leq s_{2};$$

(1-3) $$\tau =\tau_{2},s>s_{2}$$

where

α—the ultimate bond strength power.

p—softening coefficient of the descending section, obtained by fitting the test data.

$\tau_{1}$,$s_{1}$—the ultimate bond strength and the corresponding slip (pull-out displacement) values.

$\tau_{2}$,$s_{2}$—the residual bond strength and the corresponding slip values.

In 1997, Cosenza, Manfredi, Realfonzo et al. [28] presented a new model for the ascending segment of the curve because most of the structures were designed considering practical applications. Only the τ–*s* ascending segment curve was required to be more accurate. The Cosenza–Manfredi–Realfonzo (CMR) model has a more straightforward form, and the initial slope is infinity following the experimental phenomenon. However, the model did not consider the descending and residual segments of the τ–*s* curve, which has some limitations that are not applicable to some members requiring complete process analysis.

The CMR model is expressed as follows:

(1-4) $$\frac{\tau }{\tau_{1}}={\left[1-e^{-\frac{s}{\alpha }}\right]}^{\beta },s\leq s_{1}$$

where

$\tau_{1}$—the ultimate bond strength.

α,β—parameters determined by experiment.

In 2003, Gao et al. [29] proposed the continuous curve model to overcome the aforementioned problems. The model is derived in both the mathematical and physical senses based on the three critical points of the τ–*s* curve. Its physical concept is smooth and continuous at the extremum. The only problem is the residual segment, which cannot reflect the process of fluctuation decay.

The continuous curve model is expressed as follows:

(1-5) $$\frac{\tau }{\tau_{1}}=2\sqrt{\frac{s}{s_{1}}}-\frac{s}{s_{1}},0<s\leq s_{1}$$

(1-6) $$\tau =\tau_{0}\frac{{\left(s_{2}-s\right)}^{2}(2s+s_{2}-3s_{1})}{{\left(s_{2}-s_{1}\right)}^{3}}+\tau_{2}\frac{{\left(s-s_{1}\right)}^{2}(3s_{2}-2s-s_{1})}{{\left(s_{2}-s_{1}\right)}^{3}},s_{1}<s\leq s_{2}$$

where

$\tau_{1}$,$s_{1}$—the ultimate bond strength and the corresponding slip values.

$\tau_{2}$,$s_{2}$—the residual bond strength and the corresponding slip values.

In 2009, Hao et al. [30] established a bond–slip constitutive model between GFRP and steel strand composite bars and concrete, taking into account the process of gradual cyclic decay of the residual segment and fitting it. However, the formula had many uncertain parameters, which were more difficult to handle.

The model is expressed as follows:

(1-7) $$\tau =\tau_{1}(\frac{s}{s_{1}}),0<s\leq s_{1}$$

(1-8) $$\tau =(\tau_{2}-\tau_{1}){\left[\frac{\left(s-s_{1}\right)}{\left(s_{2}-s_{1}\right)}\right]}^{\alpha }+\tau_{1},s_{1}<s\leq s_{2}$$

(1-9) $$\tau =\tau_{2}[1-\beta (\frac{s}{s_{2}})-1],s_{2}<s\leq s_{3}$$

(1-10) $$\tau =\tau_{3}-\gamma [e^{-\xi \omega (s-s_{3})}\cos\omega (s-s_{3})-1]+\rho (e^{-\xi \omega (s-s_{3})}-1),s>s_{3}$$

where

$\tau_{1},\tau_{2},\tau_{3}$—the microslip, ultimate, and residual bond strengths, respectively.

$s_{1},s_{2},s_{3}$—the corresponding slip values.

α,β,γ,ξ,ω,ρ—parameters determined by experiment.

In 2019, Chen et al. [31] improved the previous theory and proposed a bond–slip constitutive model of FRP with coral concrete. A sinusoidal decay function was first used to simulate the bond degradation process of the residual segment while satisfying the condition of the infinite initial slope and continuously smooth extremum. However, the simultaneous consideration of the descending and residual segments leads to a fitting error in the case of a large slope of the curve of the descending segment.

The Chen model is expressed as follows:

(1-11) $$\frac{\tau }{\tau_{1}}=2{\left(\frac{s}{s_{1}}\right)}^{\frac{1}{\alpha }}-\frac{s}{s_{1}},s\leq s_{1}$$

(1-12) $$\tau =\tau_{1}-\frac{\Delta \tau }{2}[e^{\frac{\pi }{\beta }}-e^{\left(\frac{\pi -s+s_{1}}{\beta }\right)}\sin(2\pi \frac{s-s_{1}}{\Delta s}+\frac{\pi }{2})],s\geq s_{1}$$

where

$\tau_{1}$,$s_{1}$—the ultimate bond strength and the corresponding slip values.

Δτ—the bond strength between the maximum and minimum values of the residual segment.

Δs—the rib spacing of FRP bars.

α,β—parameters determined by FRP type.

The presented literature review emphasizes the continuous development of the current bond–slip models. Moreover, the literature review highlights the drawbacks and shortcomings of these models, which can be summarised as follows: (1) The research objectives of the previous studies were mainly focusing on FRP bars and rods with circular sections, whose bond–slip curves differ from CFRP strands with seven-wired geometry. (2) The descending section of the τ–*s* curve in these models is primarily straight and relatively smooth.

In this study, the bonding behaviour of CFRP strands in prestressed concrete is investigated and compared to the conventional steel strands. CFRP bars are more suitable for prestressed concrete strands and anchor cables due to their excellent corrosion resistance and mechanical properties. However, the CFRP is relatively expensive and its elongation at break is low. In contrast, GFRP and BFRP have relatively lower prices and higher elongation at break, but their mechanical and long-term properties are not as good as CFRP, which may not be applicable to prestressed concrete structures [32,33,34]. In this research, the bond strength of CFRP strands was experimentally studied by standard pull-out tests of the anchor-inserted part of the strand filled with grouting admixture. The effect of surface treatment and coating on the bond performance of CFRP strands is also investigated for different types of concrete to alleviate the lack of research on CFRP strands–concrete bonding behaviour. In addition, the bond stress–slip model between CFRP strands and concrete was characterised and compared to steel strands. The effect of bond design on several structural parameters, such as the failure mode and bond strength, was analysed. Finally, the study proposed bond–slip constitutive models for CFRP strands with better applicability using an exponentially damped sine function to fit the residual segment of the curve. The experimental data obtained from this study add to the knowledge base needed to develop design models and guidelines for predicting the bond performance of CFRP strands with grouting admixture under direct pull-out conditions, thus widening the use of CFRP strands in prestressed concrete structures with more confidence.

## 2. Experimental Program

CFRP strands have different structures and properties than steel, such as their non-corrosive and rusting nature [35,36], high tensile strength (about four times) [37,38], lightweight nature and lower life cycle costs [39,40], and impressive fatigue resistance [41,42], which supports the limited deformation of concrete [35,36,43]. These different properties lead to different bond–slip characteristics of CFRP strands compared to steel strands. The experimental program undertaken in this study aimed to investigate the local bond–slip behaviour with different types of strands embedded in grouting admixture by performing pull-out tests for different surface treatment forms and materials.

### 2.1. Materials

#### 2.1.1. Strands

**Figure 2:**
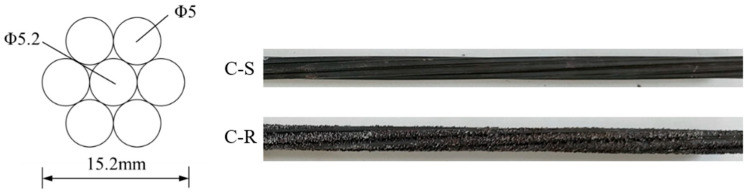
Cross-sectional dimensions and side profile of strands for testing.

In this experiment, CFRP strands with a nominal diameter of 15.2 mm and an effective cross-sectional area of 137.44 mm^2^ were produced by Zhongfu Carbon Core Cable Technology Co., Jiangsu, China to be tested. The strands were divided into two groups depending on the surface treatment: smooth strands (untreated) (C-S) and rough strands (coated with epoxy resin mortar) (C-R), as depicted in Figure 2. A mixture of epoxy resin and quartz sand was used to cover the surface of the rough specimens. The manufacturer provided the material test results, which were obtained by averaging the results of five tests. These mechanical properties are presented in Table 1.

The steel strands used in the experiment had the exact dimensions as the CFRP strands, and their mechanical properties were in accordance with ISO 6934-4-2020 [44].

#### 2.1.2. Grouting Admixture

In this experiment, a water-to-cement (*w/c*) ratio of 0.12 was chosen for the cementitious grout dry admixture. Three specimens of 40 × 40 × 160 mm were prepared by standard molds based on ISO 679-2009 [45] to determine the mechanical properties of the grouting admixture. According to GB/T 50448-2015 [46], the grouting admixture should use non-vibration molding when pouring. The mixed slurry was directly filled into the molds, then flushed with the top edge.

**Figure 3:**
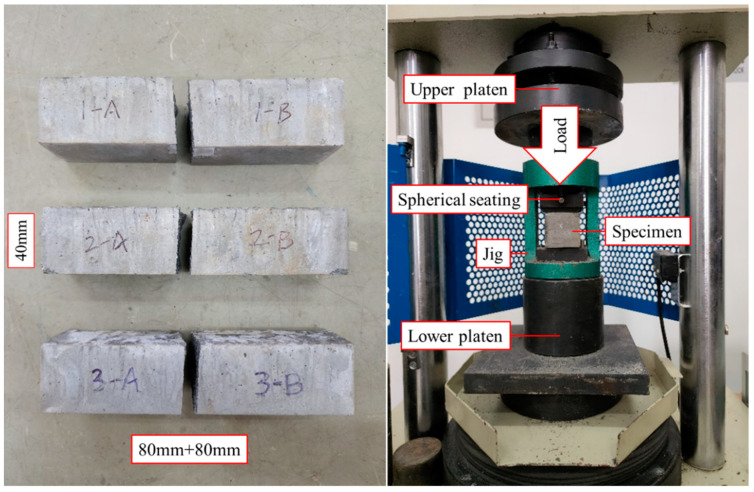
Test specimens and setup of grouting admixture material.

Steam curing was used to shorten the experimental time. The strength can reach more than 95% after 48 h of steam curing, based on the results of previous material property tests. When the specimens reached the required strength by steam curing for 48 h, the material test specimens were taken out of the constant-temperature steam-curing equipment with pull-out test specimens together. Afterwards, the flexural strength test was carried out by the flexural machine. Then the compressive strength test was carried out for each section. The specification ISO 679-2009 [45] mentions the following procedure: At the required age, the specimens are taken from their wet storage and broken in flexure, determining the flexural strength where required, or broken using other suitable means that do not subject the prism halves to harmful stresses, and each half is tested for strength in compression. After completing the flexural strength test, each specimen are divided into two parts in the bending experiment, and the separated specimens are labeled as groups A (1 to 3) and B (1 to 3) for ease of recording in subsequent compression experiments. The test specimens, setup, and results are shown in Figure 3 and Table 2. The results show that the flexural and compressive strengths of the grouting admixture used in the experiment were about 7.1 MPa and 105.3 MPa, respectively.

If one result within the six individual results varies by more than ±10% from the mean, then it should be abandoned, and the arithmetic mean is then calculated for the five remaining results according to the specification.

### 2.2. Test Setup

#### 2.2.1. Preparation of Experiments

The bond test of different types of strands and grouting admixture was designed according to ASTM D7913/D7913M−14 [47]. In the current study, bond test methods were divided into two main categories: pull-out and beam tests. The first category was mainly used to measure the bond strength and bond anchorage properties of different materials and concrete. The second test was used to explore the design strength of the bonded anchorage and related structural requirements. The test specimens of the pull-out test are relatively easy to produce and can reflect the full range of forces in the concrete.

The straightforward mechanism of the pull-out test reflected the law of force in concrete comprehensively and minimized the influence of external factors on the test results. Therefore, the pull-out test was used to study the bond strength performance.

**Figure 4:**
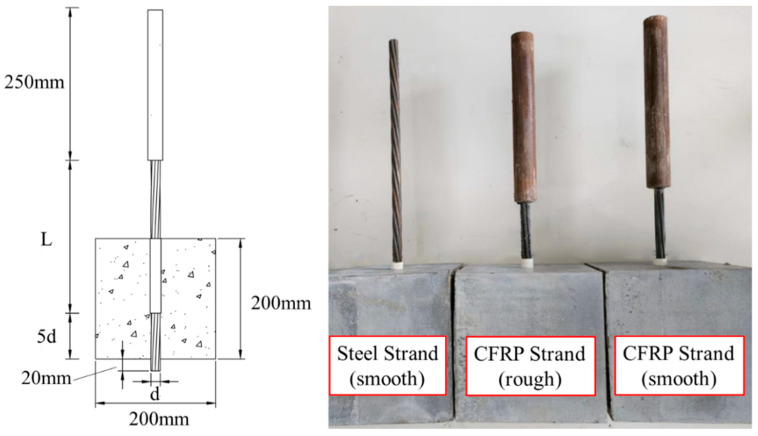
The setup and dimensions of the pull-out test specimens.

The pull-out tests used CFRP and steel strands with a total length of 600 mm. The size of each specimen was a 200 × 200 × 200 mm cubic block, with the bond length five times the strand’s diameter shown in Figure 4. Large specimens were used to avoid premature grouting-admixture splitting damage, leading to failure, which is in accord with practical application scenarios.

In order to avoid slippage during the loading process of CFRP strands, a 250 mm length female anchor barrel with an inner diameter of 25 mm was customized. When anchoring the strand, it was necessary to break the stranded wire into a monofilament to increase the contact area, thereby increasing the adhesion between the stranded wire and the sleeve. The bonding material adopted ultra-high-performance concrete dry mix (UHPC-GJL), which has ultra-high compressive performance that can prevent the end anchoring material from being crushed and broken. There was no slippage during the follow-up test due to the reliable anchoring effect.

Holes were punched on both sides of the mold with 16 mm in diameter for one side of the hole, which is slightly larger than the diameter of the strands. The other side was 18 mm in diameter with a bond breaker (plastic pipes) so that the loaded end of the specimen had a length (L) of 1200 mm. The strands passed through the mold with 20 mm exposed length outside the hole on the free end.

**Figure 5:**
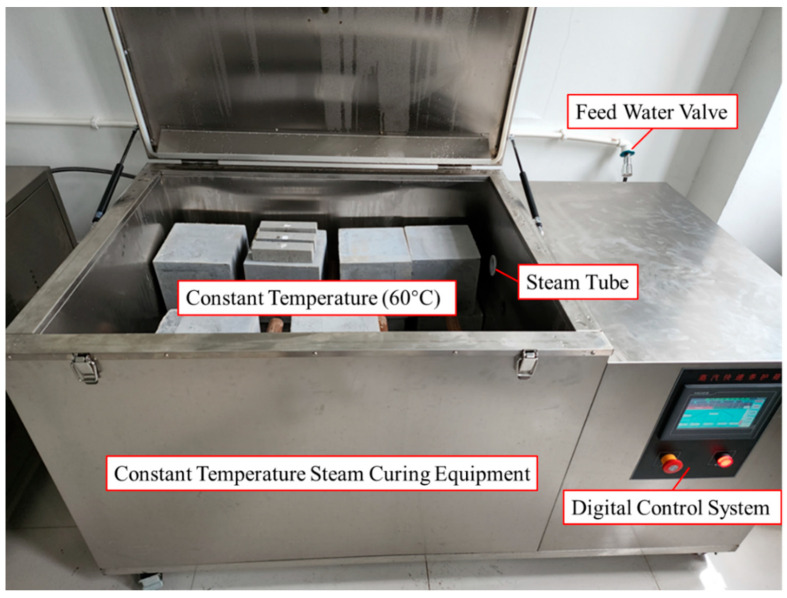
Curing environment setup for preparation of specimens.

Quick-drying ceramic mud was fixed in the gap between the pipe and stranded anchor section to prevent the strand from touching the grouting admixture. Non-vibration molding was used during the pouring process. The mold was removed after curing for 48 h at room temperature. Afterwards, the specimens were placed in the constant-temperature steam-curing equipment at 60 degrees for 48 h, as shown in Figure 5. Finally, the specimens were placed to rest at room temperature until the loading test started.

The specimen ID and main parameters are shown in Table 3. The first letter in the specimen ID represents the material type, while the second letter refers to the surface treatment. For instance, C in “C-S” represents CFRP as the strand material, and S indicates that the surface treatment is smooth (untreated). Each group of specimens had three strands.

#### 2.2.2. Experimental Procedure

The pull-out test was carried out using an MTS-1000 universal testing machine connected to a data acquisition system to record the bonding strength of strands. The slip displacement of the strands was measured by a displacement meter, and the data were collected by a DH5921 dynamic acquisition box produced by Donghua Testing Technology Co. Ltd. with the reading frequency set to 10 Hz.

**Figure 6:**
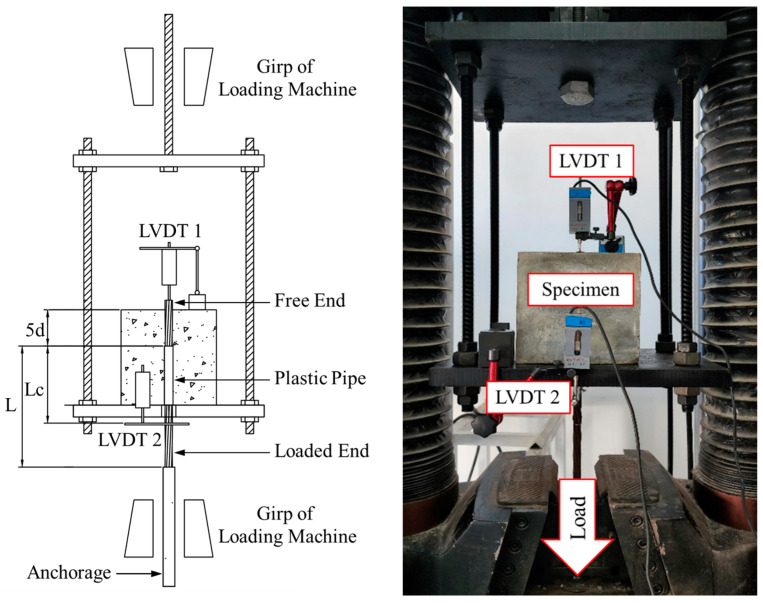
Test setup of the pull-out experiment on CFRP and steel strands.

The test was undertaken using a displacement control loading with a loading rate of 0.8 mm/min. The test setup and loading device are shown in Figure 6. During the test, the specimen was placed in the pre-customised reaction frame and a force transducer was used to measure the reaction force. At the same time, the elongation gauges 1 and 2 (LVDT 1 and 2, respectively) were installed at the loaded and free ends of the strand to measure the relative slip displacement accurately.

## 3. Results and Discussion

The bonding performance of the tested strands was evaluated in terms of the failure mode, ultimate bond strength, slip displacement, and bond stress–slip curves, as presented in the following sections.

### 3.1. Failure Modes

The bonding force between strands and concrete include two main components of (i) chemical adhesion between the cementitious gel in the concrete and the surface of the strand, and (ii) friction between the contact surface of the strand and grouting admixture [48]. In the current study, the considered breakage mode of the tested strands was only a pull-out failure mode.

**Figure 7:**
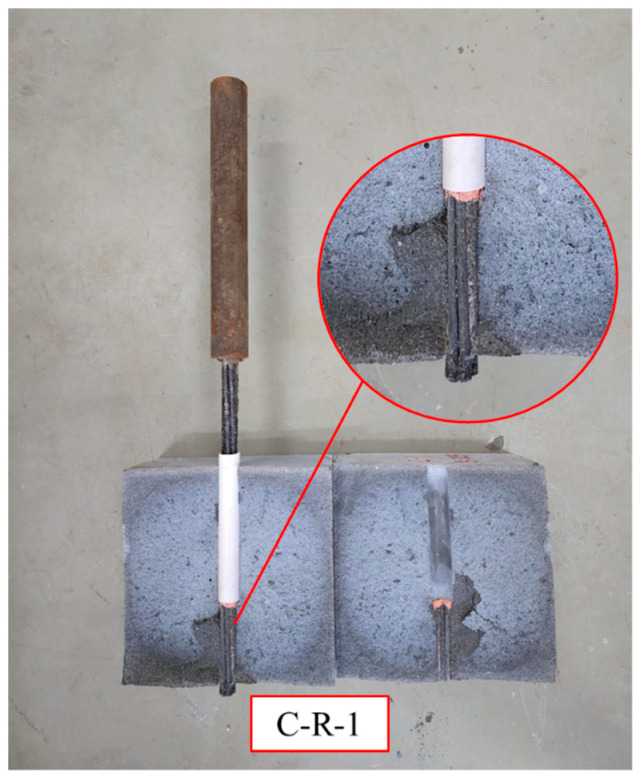
The pull-out failure mode of C-R-1 specimen.

At the beginning of loading, the bonding force between the strands and the concrete was caused mainly by the chemical adhesion component, which represents the reactions that occur during concrete curing as the (i) of the bonding force. As the loading increased, the bond force decreased gradually and the mechanical bite force and friction force increased gradually, causing wear on the surface of the strands due to friction. The observed wear reduced the mechanical bite force further until the strands were pulled out. When the test setup was pulled out, the strands were pulled out from the adhesive medium, and the surface of the strands was severely worn. The surface of the strand with sticky sand was completely peeled off, as shown in Figure 7, when splitting the grouting admixture after the test.

**Figure 8:**
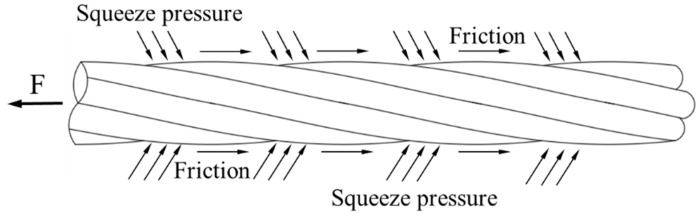
CFRP strand under mechanical pressure and friction force.

During the experiment, the grouting admixture was mainly subjected to the mechanical force and friction generated by the CFRP strand surface, as shown in Figure 8. When the pull-out force increased, the grouting admixture started to crack in front of the strand rib due to the oblique extrusion, and then a small internal crack was generated. When the annular tensile stress was higher than the ultimate tensile strength of the grouting admixture, the internal radial cracks expanded in the grouting admixture and continued to form longitudinal splitting cracks when reaching the surface of the protective layer. Finally, this failure mechanism led to splitting damage of the concrete. No significant grouting admixture splitting occurred in all the groups, since the surface ribs of the tested CFRP strand were not obvious and resulted in an insignificant effect of extrusion stress on the grout.

### 3.2. Ultimate Bond Strength

Referring to GB/T 50152-2012 [49], the ultimate bond strength of the tested CFRP strands and grouting admixture was measured by calculating the maximum tensile bearing capacity using Formula (3-1):

(3-1) $$\tau_{\max}=\frac{F_{\max}}{\pi ld}$$

where

$\tau_{\max}$—the ultimate bond strength.

*d*—the diameter of the GFRP strands.

*l*—the anchorage length of the GFRP bars.

$F_{max}$—maximum pull-out load capacity.

### 3.3. Slip

The slip displacement at the free end (S1) was directly measured by displacement gauge 1, while the slip at the loaded end (S2) was calculated as the following. The elongation of the strand between the displacement gauge and the anchored section was defined as Δ*S*. The length from the top of the embedded strand to the point of the attachment of the measuring device was defined as *L_C_*. The formulas used to calculate the slip at the loading end are shown in Formulas (3-2) and (3-3):

(3-2) $$S_{2}=S-\Delta S$$

(3-3) $$\Delta S=\frac{PL_{c}}{EA}$$

where

*p*—maximum load.

*S*—actual measured value of the displacement meter.

*E*—modulus of elasticity of the strand.

An average measured slip value of 1 mm at the free end was considered as a sign of losing bond capacity according to relevant research [46] on steel strands. For the tested CFRP strands, the maximum bonding force was used to calculate the bonding strength.

**Figure 9:**
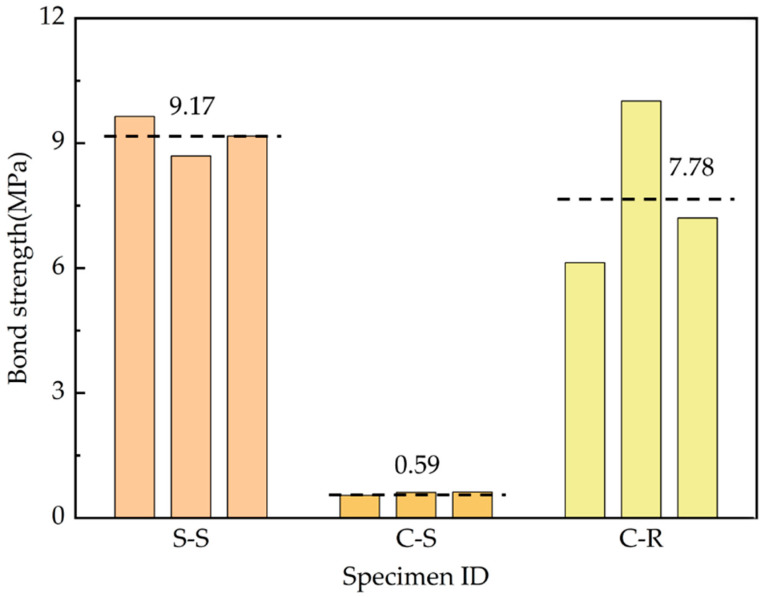
Experimental results of the pull-out test on the three different groups of specimens.

The measured results of CFRP/steel strands with grouting admixture in the pull-out tests are shown in Figure 9 and Table 4. It is evident that the bond strength of the smooth CFRP strand (C-S) is significantly lower than the other two groups of specimens. The ultimate bond strength of the steel strand (S-S) and rough CFRP strand (C-R) was similar, with the latter exhibiting a larger dispersion of values, which was referred to as the effect of sand bonding.

### 3.4. Stress–Slip Curve

**Figure 10:**
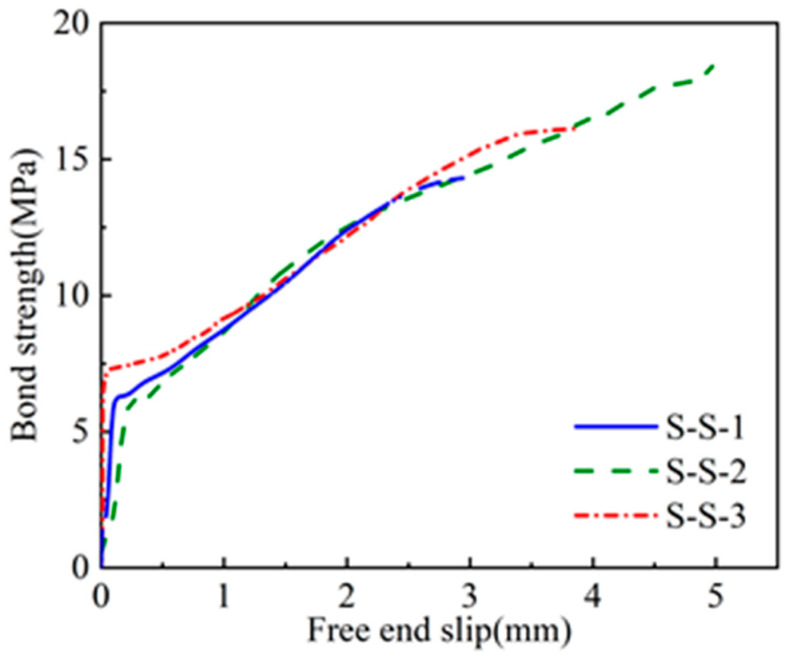
Bond–slip curves of the smooth steel strands (S-S).

**Figure 11:**
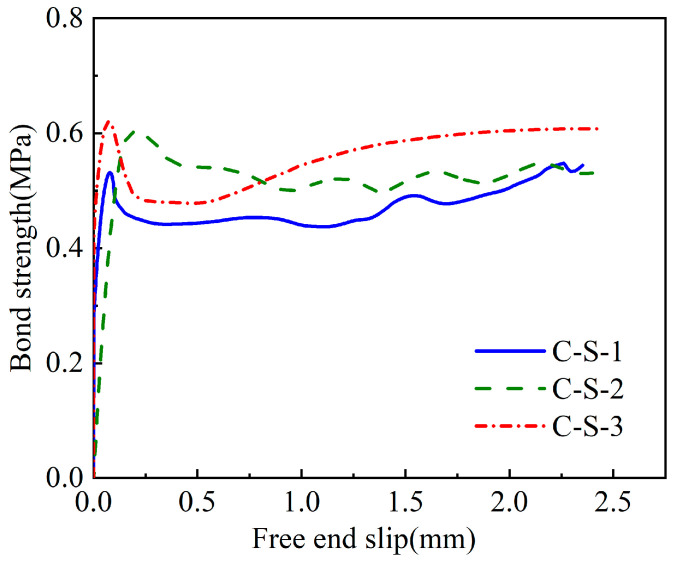
Bond–slip curves of the smooth CFRP strands (C-S).

**Figure 12:**
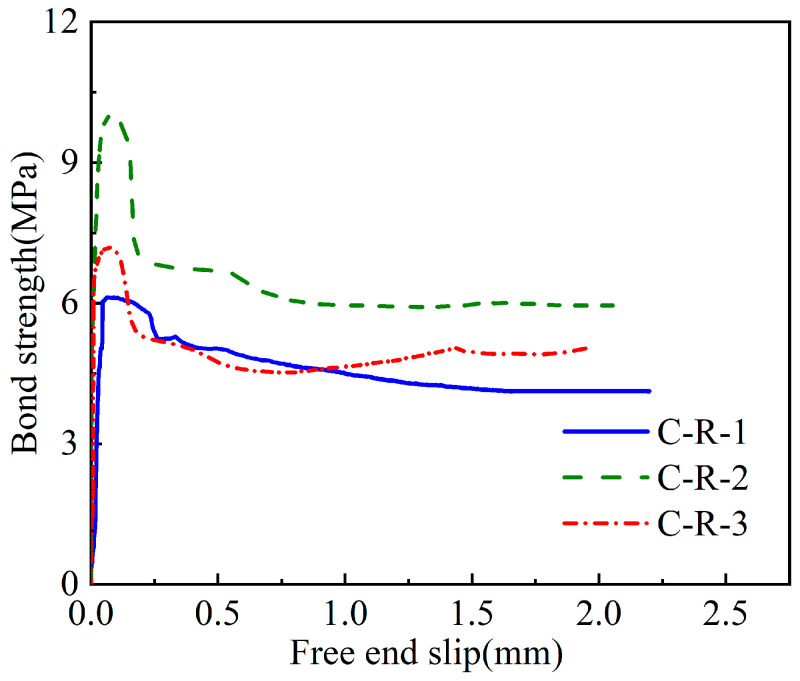
Bond–slip curves of the rough CFRP strands (C-R).

The experimental failure mode of CFRP- and steel-strand-bonded specimens was characterised as a pull-out failure, as discussed in Section 3.1. Figure 10, Figure 11 and Figure 12 show the bond strength–slip (τ–*S*) curves of the three different groups of strands investigated in this study.

It was found that the τ–*S* curves of CFRP strands have different patterns for different damage modes compared to steel strands. When pull-out damage occurs, the τ–*S* curve of CFRP strands can be divided into ascending, descending, and residual segments, among which the ascending segment can be divided into microslip and slip segments. On the other hand, the damage mode of the steel-strands-bonded specimens was all pull-out damage, and the τ–*S* curve was still rising for a period of time after the strand was pulled out.

The bond–slip relationship between CFRP stranded wire and grout was different from that of steel strands, and the τ–*S* curve had different morphologies at different loading stages, as shown in Figure 10, Figure 11 and Figure 12. When the specimen was pulled out and damaged, the bonding–slip curve was characterised by a rising section, falling section, and residual section, among which the rising section was divided into microslip section and slip section. The failure mode of steel strand specimens was pull-out failure, and the τ–*S* curve continued to rise at a relatively stable rate after slipping. The following points describe the stages present in the bond–slip curve of the tested specimens:

(i) Microslip section. At the beginning of the specimen loading, the bonding stress gradually decreases along the anchorage length from the specimen loading end to the free end. At this time, the internal chemical adhesion of the specimen has not disappeared yet and the slippage of the loading end of the specimen is not yet obvious. Generally, the free end does not slip and the bonding–slip curve shows a vertical rise at this time.
(ii) Slip section. After the microslip section, the free end of the specimen begins to slip. However, the rate of change is still lower than that of the loading end, and the bond–slip curve increases linearly at this time. For sticky sand CFRP stranded wires, there is also a large amount of chemical adhesion to keep the free end slip-stable due to the good bonding between quartz sand and concrete.
(iii) Descending section. When the bonding stress reaches its limit, the load drops abruptly, accompanied by the crushing sound of concrete. At this time, the rate of change between the free end and the loading end is close and the bond–slip curve has an obvious deflection or decrease. Steel strands show a change in the slope of the curve while both groups of CFRP strands show a curve decline.
(iv) Residual segment. At this stage, the specimen slips significantly and the slippage of the two detection points is increased rapidly. For steel strands, the bond–slip curve fluctuates upward, while CFRP strands fluctuate up and down around a constant value.

**Figure 13:**
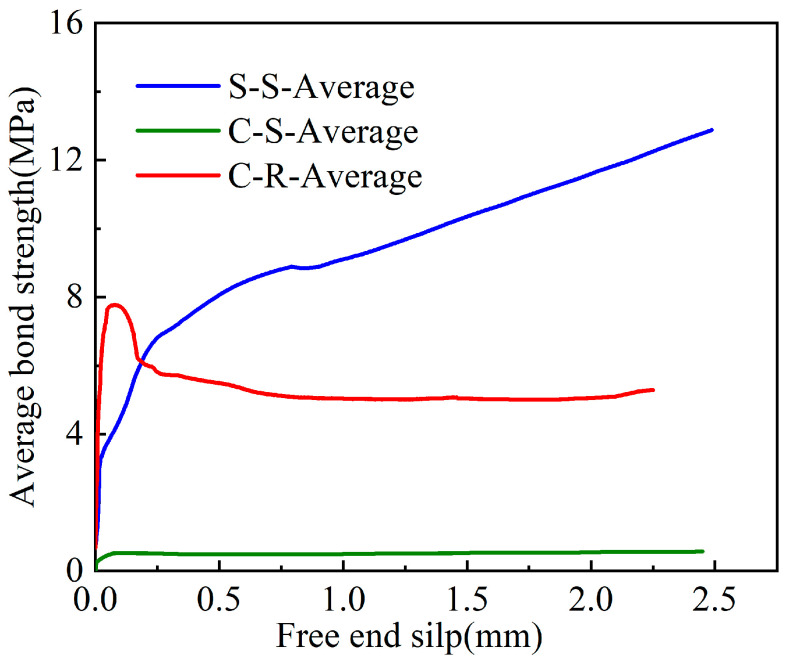
Average bond–slip curves of the three different strands groups (S-S\C-S\C-R).

The average bond strength–slip curves of the three groups of specimens are shown in Figure 13 in order to depict the different curve stages of each group. The average curve is obtained by calculating the mean value of multiple curves in Origin software to make the fitting curve more representative.

From Figure 13, the following observations were noticed:

(i) First, the steel bar had less discreteness than FRP, and the ultimate strength difference of the specimens in the S-S was not more than 10% (7.1, 6.3, and 6 MPa). The ultimate strengths of the specimen curves of the C-S and C-R were quite different, but the trends of the curves were consistent.
(ii) From the average curves, the ultimate strengths of S-S and C-R were close to (7.5–8 MPa), which are much greater than the 0.8 MPa of C-S. This indicates that the bonding strength of the surface-treated C-R specimen is close to the smooth steel bar used in the actual project, and has excellent bonding performance.
(iii) The slip (at the end) was much smaller in the C-S and C-R cases (~0.1 mm) compared to the S-S case (~0.75 mm). This means that the displacement of CFRP before reaching the ultimate bond strength is small, which has certain advantages for practical engineering applications.

## 4. Bond Stress–Slip Model

After characterising the bonding behaviour of CFRP strands for prestressed concrete experimentally, a bond stress–slip constitutive model is proposed in this section to establish an analytical solution for accurate design guidelines and standards to be prepared in future. This model is developed and verified in the following subsections.

### 4.1. Development of the Bond–Slip Constitutive Model

During the experiment, the complete bond–slip response of CFRP strands and grouting admixture was collected in order to accurately reflect the effect of loading and failure behaviours. Due to the special structural morphology of CFRP strands, there was a need to find a suitable bond–slip model between the CFRP strand and grouting admixture with the following characteristics:

(i) The slope of the ascending segment is more significant, and there is almost no slip at the free end.
(ii) Once slip occurs, there is a significant decrease in bond strength.
(iii) The attenuation of the residual segment is not apparent. This observation highlights the shortfall of the existing models to fully capture the bond relationship between CFRP strands and grouting admixture.

**Figure 14:**
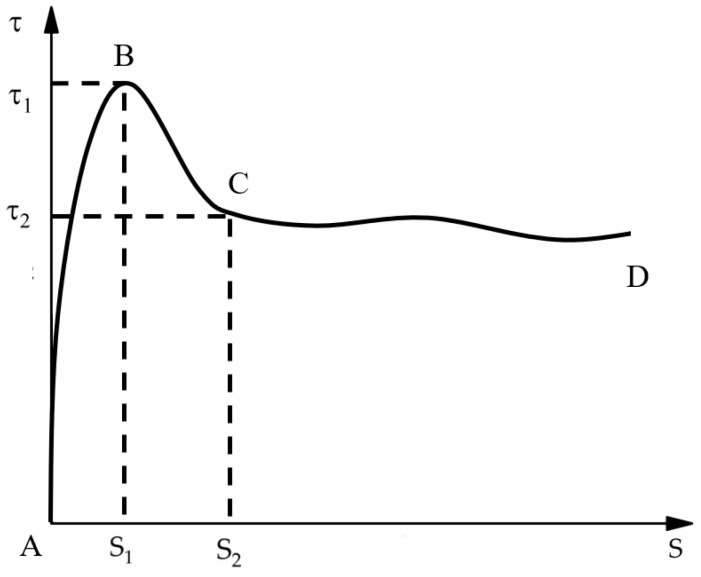
Average bond stress–slip curves of different CFRP strands.

The required constitutive model curves include three key points of A, B, and C, as shown in Figure 14. The AB section of the τ–*s* curve represents the ascending segment, with τ = 0 and dτ/ds = ∞ at point A. The BC section represents the descending segment, with τ = τ1 and dτ/ds = 0 at point B. Finally, the CD section represents the residual segment, which is a gradual cyclic decay process.

The experimental results obtained from this study can be used to verify the current bond–slip constitutive model and provide adjustments to establish a more accurate model. After comparing the experimental bond–slip curves with the available models [26,27,28], it was found that the continuous curve model can better simulate the ascending segments and descending segments of τ–*s* curves between FRP bars and grouting admixture. Moreover, this model satisfies the requirements of the clear physical concept of the formula and smooth–continuous curve

However, as we can see from the Figure 1 and Formulas (1-1)–(1-11) that the existing models are almost linear after exceeding the S2 point. These models fail to accurately describe the residual segment due to the large fluctuation error and expression difficulty. Experimentally, the residual segment between the CFRP strand and grouting admixture was found to have a periodic pattern. However, the existing model is more complicated and there is a certain error in the fitting process in this case. Therefore, adjustments were proposed in this study to correct the model based on the experimental results in order to make the expression formula more accurate.

According to the experimental results, the bond strength of the residual segment decays cyclically with the increase in slip, and the change in the *Y* axis direction is not significant. Thus, the decay process after the bond failure between the CFRP strand and grouting admixture is simulated by using the sinusoidal decay function. The expression of the residual segment function was obtained by comprehensive analysis of the experimental data with appropriate corrections. The final obtained intrinsic model that can describe the complete response of bon–-slip is shown as follows:

(4-1) $$\frac{\tau }{\tau_{1}}=2{\left(\frac{s}{s_{1}}\right)}^{\alpha }-\frac{s}{s_{1}},s\leq s_{1}$$

(4-2) $$\tau =\tau_{0}\frac{{\left(s_{2}-s\right)}^{2}(2s+s_{2}-3s_{1})}{{\left(s_{2}-s_{1}\right)}^{3}}+\tau_{2}\frac{{\left(s-s_{1}\right)}^{2}(3s_{2}-2s-s_{1})}{{\left(s_{2}-s_{1}\right)}^{3}},s_{1}<s\leq s_{2}$$

(4-3) $$\tau =\tau_{2}-\beta \tau_{2}\left[-e^{1-s}\sin(\pi \left(s-s_{2}\right)+\frac{\gamma }{2}\pi \right)],s>s_{2}$$

where

$\tau_{1}$,$s_{1}$—the ultimate bond strength and the corresponding slip values.

$\tau_{2}$,$s_{2}$—the residual bond strength and the corresponding slip values.

α,β,γ—variable parameters determined by fitting the original data point.

### 4.2. Model Verification

In practical applications (e.g., calculating the anchorage length of tendons), the ascending segment of the curve is essential and the related studies are adequate. Nevertheless, the existing models are often described by linear functions for the descending and residual segments, which are too simple. Thus, this study compared only the ascending segments of the model.

**Figure 15:**
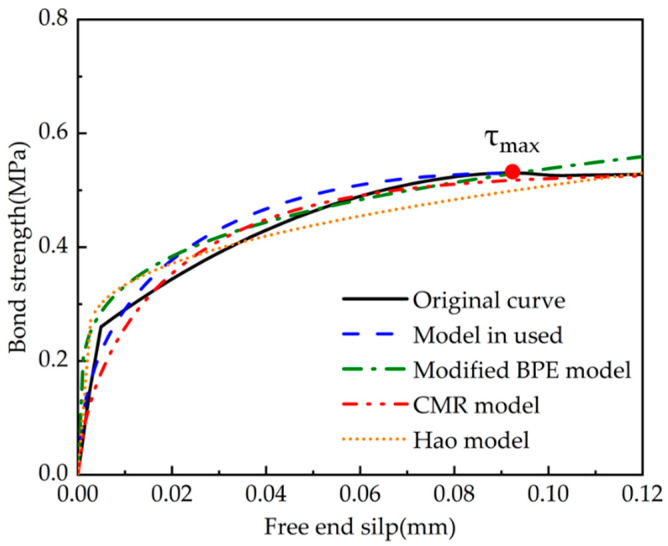
Comparison of fitting bond–slip curves for smooth CFRP strands (ascending segment).

**Figure 16:**
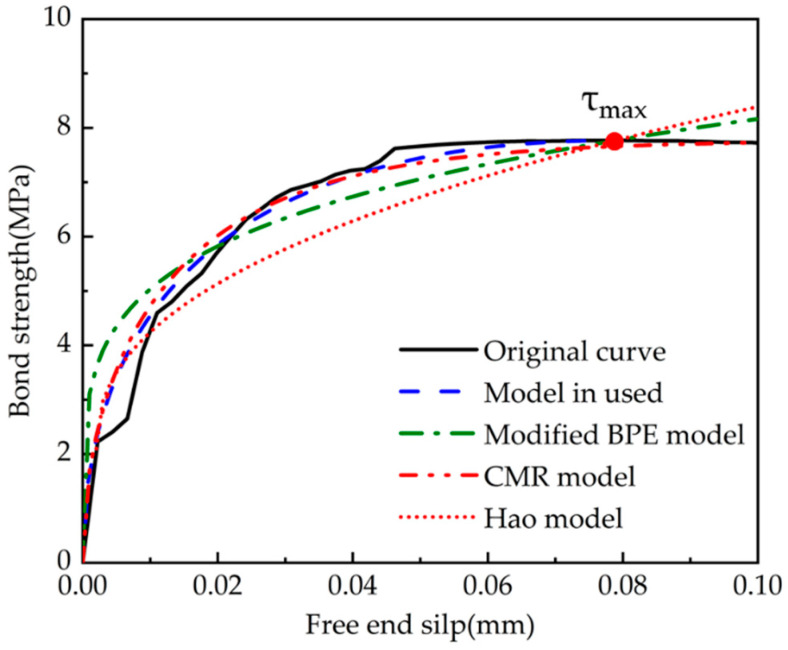
Comparison of fitting bond–slip curves for rough CFRP strands (ascending segment).

The theoretical bond–slip curves were obtained by implementing each set of test parameters into the Formula (4-1) and comparing them with the ascending segment of average bond strength–average slip curves for each stage of the experiments, shown in Figure 15 and Figure 16. The comparison model selected the modified BPE model, the CMR model, and the model proposed by Hao et al. The solid line represents the actual test data, and the dashed line refers to the calculation of the intrinsic model. The specific parameters of each fitting curve formula are shown in Table 5 and Table 6, which show the comparison of the parameters and the R-squared in order to reflect the fitting dispersion from the original curve.

From the generated figures, it was found that the slopes of the modified BPE model and the Hao model were not zero at the maximum bond strength as discussed in the literature review on the advantages and disadvantages of each model. This finding emphasises the deviation of the discussed models from the actual test results. The CMR model and the model used in this paper can better describe the rising section of the τ–*s* curve, which agrees well with the experimental results. From the generated tables, it is evident that the model used in this paper has a better overall fit, with an R-squared close to the CMR model (this model is essentially based on the CMR model) for rough strands, and better than all other models for smooth strands.

**Figure 17:**
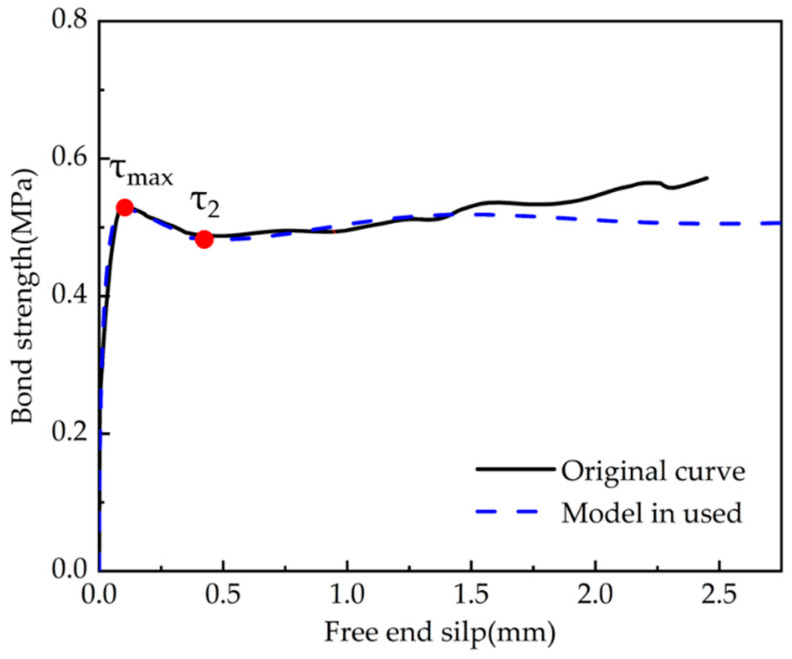
Fitted bond–slip curves versus the original curves of smooth CFRP strands.

**Figure 18:**
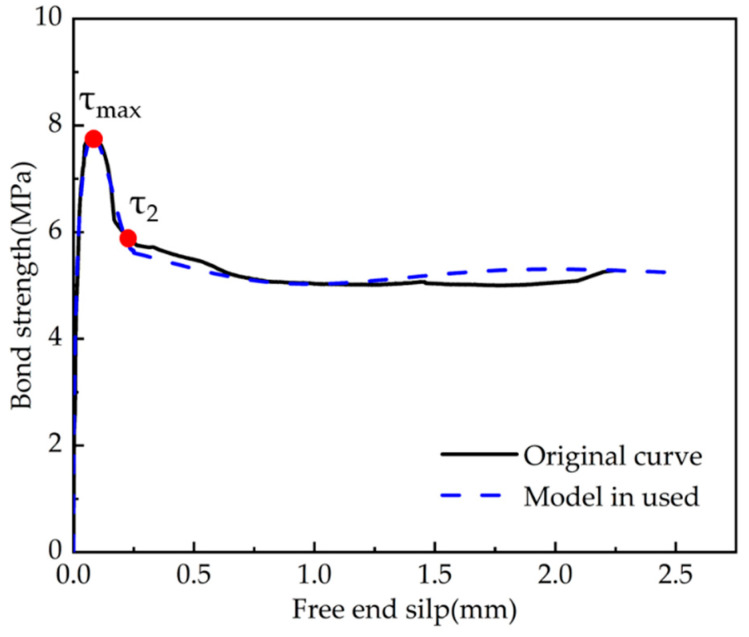
Fitted bond–slip curves versus the original curves of rough CFRP strands.

In this study, the average bond–slip original curves of the two specimen groups (C-S and C-R) were fit and divided into ascending, descending, and residual segments. Then the fitted curves were analysed by implementing them into the proposed formulation. The relevant parameters are shown in Table 7, and the fitted curves are shown in Figure 17 and Figure 18.

It is evident that the proposed model curve agrees well with each segment of the experimental bond–slip curve of the CFRP strands and grouting admixture. The comparative analysis of the generated figures leads to the following conclusions:

(i) The degree of agreement for the ascending section is higher than that for the descending and residual sections of the curve, and the fitting model for the residual section can be further improved.
(ii) The smooth CFRP strand can still rely on mechanical anchoring force to rise partly after slipping due to poor bonding performance. The current model faces some difficulties in effectively simulating the rise of the bonded residual section.
(iii) According to the fitting results, the values of the parameters are taken to be relatively close. For the parameters of the proposed formulas in text, it is suggested that $\alpha =\frac{1}{2}$, $\beta =\frac{1}{25}$, and γ=1,2.

## 5. Conclusions

In this research, an experimental study was undertaken to investigate the bond behaviour of seven-wire CFRP strands and study their composite performance for prestressed concrete. CFRP strands are different from the commonly used steel tendons due to their material construction and bonding mechanism. Thus, the bonding performance of CFRP strands as anchor cables with grouting admixture was examined and compared to steel strands in order to obtain design models and guidelines for CFRP prestressed concrete. Three groups of standard pull-out tests were conducted. The ultimate bond strength and slip displacement between the strands and grouting admixture were calculated and compared. The effect of different surface treatments on the bond strength was characterised with the epoxy quartz sand surface recording the optimal bonding performance. In addition, the bond–slip constitutive model of CFRP strands was improved based on existing models to be more accurate and consistent to fit a larger domain of the experimental data. Based on the current research, the following conclusions were obtained:

(i) Untreated CFRP strands have a fragile bond strength with the grouting admixture, equivalent to only 5% compared to steel strands of the same diameter. This type of anchorage failure was related to the smooth surface, which exhibited inefficient mechanical anchorage force.
(ii) The surface coating of epoxy quartz sand on CFRP strands significantly improved the anchoring efficiency and bonding strength by up to 14 times to achieve 77% of the bond strength when compared to steel strands.
(iii) A momentary drop in the bonding force was observed as the CFRP strand slipped out of the grouting admixture. This force represents the maximum bonding force at this point and maintains its level afterwards. The strand’s bond–slip curve was observed to still grow steadily, although its slope decreased after an evident displacement of the free end.
(iv) The failure of CFRP strands with a surface coating of epoxy quartz sand was triggered by the flaking of quartz sand, which represents the starting point to parametrize different types of epoxy and quartz sand in order to improve the bond strength of CFRP strands.
(v) Bond–slip constitutive models with better applicability were proposed to mitigate the shortage in the current bond–slip constitutive models describing the behaviour of CFRP strands and grouting admixture. The proposed constitutive models were developed based on previous studies using an exponentially damped sine function to fit the residual segment of the curve, which closely agreed with the experimental results.

## Figures and Tables

**Table 1:** Material properties of CFRP and steel strands.

Material of Strands	Tensile Strength/ MPa	Elastic Modulus/ GPa	Density/ (kg/m^3^)	Ultimate Elongation/ %
CFRP (smooth/rough)	2704	170	0.227	1.50
Steel (smooth)	1720	195	1.101	3.50

**Table 2:** Flexural and compressive strength properties of grouting admixture specimens.

Specimen ID	Specimen Part	Flexural Load (KN)	Flexural Strength (MPa)	Compressive Load (KN)	Compressive Strength (MPa)
1	A	3.06	7.17	161.76	101.1
	B			162.23	101.39
2	A	2.85	6.68	170.08	106.3
	B			176.27	110.17
3	A	3.15	7.38	146.51	91.57
	B			171.78	107.36
Average		3.02	7.08	168.44	105.26

**Table 3:** Details of test specimens.

Specimen ID	Material	Surface Treatment	Quantity
S-S	Steel	Smooth	3
C-S	CFRP	Smooth	3
C-R	CFRP	Rough	3

**Table 4:** Statistical evaluation of the experimental results of the three groups of specimens.

Specimen ID	Ultimate Pull-Out Load (KN)	Bond Strength (MPa)	Slip at Loaded End (mm)	Slip at Free End (mm)	Average Bond Strength (MPa)	Coefficient of Variation (COV)
S-S-1	34.96	9.64	1.88	0.99	9.17	0.042
S-S-2	31.51	8.69	4.52	1.02		
S-S-3	33.25	9.17	2.63	1.00		
C-S-1	1.95	0.54	0.45	0.05	0.59	0.002
C-S-2	2.22	0.61	0.31	0.19		
C-S-3	2.24	0.62	0.92	0.02		
C-R-1	22.22	6.13	1.22	0.085	7.78	0.344
C-R-2	36.34	10.01	1.27	0.08		
C-R-3	26.11	7.20	1.99	0.066		

**Table 5:** Comparison of fitting bond–slip curve parameters (ascending segment).

Model ID	α	β	R^2^
Model used	0.54	-	0.991
Modified BPE model	0.28	-	0.985
CMR model	0.034	0.49	0.987
Hao model	0.50	-	0.876

**Table 6:** Comparison of fitting bond–slip curve parameters (ascending segment).

Model ID	α	β	R^2^
Model used	0.51	-	0.980
Modified BPE model	0.25	-	0.899
CMR model	0.016	0.80	0.990
Hao model	0.33	-	0.899

**Table 7:** Comparison of fitting bond–slip curve parameters (residual segment).

Specimen ID	$\tau_{1}$	$s_{1}$	$\tau_{2}$	$s_{2}$	β	γ	R^2^
C-S Average	0.59	0.093	0.51	0.37	0.039	1.1	0.555
C-R Average	7.78	0.079	5.23	0.25	0.044	2.1	0.601

## Data Availability

Not applicable.
